# Diffusion-Based Causal Representation Learning

**DOI:** 10.3390/e26070556

**Published:** 2024-06-28

**Authors:** Amir Mohammad Karimi Mamaghan, Andrea Dittadi, Stefan Bauer, Karl Henrik Johansson, Francesco Quinzan

**Affiliations:** 1 Division of Decision and Control Systems (DCS), KTH Royal Institute of Technology, 114 28 Stockholm, Sweden; kallej@kth.se; 2 Helmholtz AI, 85764 Munich, Germany; andrea.dittadi@helmholtz-munich.de (A.D.); stefan.bauer@helmholtz-munich.de (S.B.); 3 MPI for Intelligent Systems, 72076 Tübingen, Germany; 4 School of Computation, Information and Technology, TU Munich, 80333 Munich, Germany; 5 Digital Futures, 114 28 Stockholm, Sweden; 6 Department of Computer Science, University of Oxford, Oxford OX1 2JD, UK; francesco.quinzan@cs.ox.ac.uk

**Keywords:** diffusion models, diffusion-based representations, causal representation learning, weak supervision

## Abstract

Causal reasoning can be considered a cornerstone of intelligent systems. Having access to an underlying causal graph comes with the promise of cause–effect estimation and the identification of efficient and safe interventions. However, learning causal representations remains a major challenge, due to the complexity of many real-world systems. Previous works on causal representation learning have mostly focused on Variational Auto-Encoders (VAEs). These methods only provide representations from a point estimate, and they are less effective at handling high dimensions. To overcome these problems, we propose a Diffusion-based Causal Representation Learning (DCRL) framework which uses diffusion-based representations for causal discovery in the latent space. DCRL provides access to both single-dimensional and infinite-dimensional latent codes, which encode different levels of information. In a first proof of principle, we investigate the use of DCRL for causal representation learning in a weakly supervised setting. We further demonstrate experimentally that this approach performs comparably well in identifying the latent causal structure and causal variables.

## 1. Introduction

Causal representation learning consists of uncovering a system’s latent causal factors and their relationships, from observed low-level data. It finds applicability in domains such as autonomous driving [1], robotics [2], healthcare [3], climate studies [4], epidemiology [5,6], and finance [7]. Furthermore, recent advancements in Large Language Models (LLMs) underscore the growing importance of studying causal representation learning in this domain [8,9,10]. In these tasks, the underlying causal variables are often unknown, and we only have access to low-level representations.

Causal representation learning is a challenging problem. In fact, identifying latent causal factors is generally impossible from observational data only. There has been an ongoing effort to study sets of assumptions that ensure the identifiability of causal variables and their relationships [1,11,12,13,14,15,16,17]. These approaches consider the availability of additional information, or they use assumptions on the underlying causal structure of the DGP. However, many of these assumptions, such as Causal Faithfulness [18] cannot be verified. However, it is possible to identify latent causal factors from observational *and* interventional data. Brehmer et al. [14] considers a weak form of supervision, in which we have access to a data pair, corresponding to the state of the system before and after a random unknown intervention. Brehmer et al. [14] proves that, in this weakly supervised setting, the structure and the causal variables are identifiable up to a relabeling and elementwise reparameterization.

There has been a growing interest in leveraging generative models to learn causal representations with specific properties. For example, disentangled and object-centric representations have been shown to be helpful for complex downstream tasks and generalization [19,20,21,22,23,24]. Variational Autoencoders (VAEs) [25] are among the most widely studied generative models, and they have been successfully used for disentanglement and causal representation learning [14,26]. However, the problem of learning causal representations has not yet been approached with more powerful generative models.

Recently, diffusion models have emerged as state-of-the-art generative models, and they have demonstrated remarkable success across several domains such as image, video, and audio synthesis [27,28,29,30,31,32,33,34,35,36,37], molecular generation [38,39,40,41], and representation learning [42,43,44,45,46,47,48]. Diffusion models draw on concepts and principles from diffusion processes to learn the data distribution [49,50,51,52,53]. These models exploit diffusion behavior to produce diverse, high-quality, and realistic samples. Furthermore, unlike other generative models like VAEs that encode the information in one single code, diffusion-based models have the appealing property of infinite-dimensional latent codes which contain different levels of information at different timesteps [43]. However, despite this advantage and their remarkable performance, diffusion models have not yet been employed for causal representation learning, indicating that their potential has yet to be explored in this context.

In this work, we study the connection between diffusion-based models and causal structure learning by employing representations obtained from diffusion models for the task of causal representation learning. In particular, our contributions are the following:

- We propose *DCRL*, a diffusion-based framework for causal representation learning in weakly supervised settings.
- We derive the Evidence Lower Bound (ELBO) for DCRL, in the case of both finite- and infinite-dimensional representations.
- We empirically illustrate that the noise- and diffusion-based representations contain equivalent information about the underlying causal variables and causal mechanisms, and can be used interchangeably.

The rest of the paper is organized as follows: Section 2 explains the related works. Section 3 covers the background on causality and diffusion models. The background on diffusion models and diffusion-based representations are outlined in Section 4. Section 5 outlines the addressed problem, the weakly supervised framework, and the identifiability conditions. Section 6 details the proposed DCRL framework. Experimental results are presented in Section 7. Finally, Section 8 concludes the paper and suggests potential future research directions.

## 2. Related Work

### 2.1. Diffusion-Based Representation Learning

Learning representations with diffusion models remains a relatively unexplored area. Several works have tried to train an external module (e.g., an encoder) along with the score function of the diffusion model to extract representations. Abstreiter et al. [43] and Mittal et al. [44] condition the score function of a diffusion model on a time-independent and time-dependent encoder and obtain finite and infinite-dimensional representations, respectively. Wang et al. [45] uses the same conditioning but regularizes the objective function with the mutual information between the input data and learned representations. Traub [48] performs the same conditioning but the authors use Latent Diffusion Models [54], where the inputs of the diffusion model are latent variables obtained from applying a pre-trained autoencoder on the input. Furthermore, Kwon et al. [46] proposes an asymmetric reverse process that discovers the semantic latent space of a frozen diffusion model, where modification in the space synthesizes various attributes on input images. However, in principle, diffusion models lack a semantic latent space and it is unclear how to efficiently learn representations using their capabilities.

### 2.2. Causal Representation Learning

Given the inherent challenges of identifiability in causal representation learning, many previous studies have tackled this issue by imposing certain assumptions on the dataset or the causal structure. Several previous methods rely on additional knowledge of the data generation process, such as knowledge of the causal graph or labels for high-level causal variables. CausalGAN [55] requires the structure of the underlying causal graph to be known. Yang et al. [11] and Liu et al. [12] assume a linear structural equation model, and they require additional information associated with the true causal concepts as supervising signals. Similar to Yang et al. [11], Komanduri et al. [56] assumes the availability of supplementary supervision labels but without requiring mutual independence among factors. Von Kügelgen et al. [57] investigates self-supervised causal representation learning by utilizing a known, but non-trivial, causal graph between content and style factors. Subramanian et al. [13] applies Bayesian structure learning in the latent space and relies on having interventional samples. Sturma et al. [58] considers a setup where the authors have access to data from multiple domains that share a causal representation. Buchholz et al. [59] assumes the latent distribution is Gaussian and the authors have access to unknown single-node interventional samples. Additionally, Ahuja et al. [15] analyzes various scenarios and the level of identifiability in the presence of interventional data. For an overview of causal representation learning, we refer to Schölkopf et al. [1].

Furthermore, there have been recent works on utilizing diffusion models in causality. Specifically, Sanchez and Tsaftaris [60] focuses on counterfactual estimation from observational imaging data given a known causal structure. Similarly, Sanchez et al. [61] aims to learn the underlying SCM in the low-level data space assuming a non-linear additive noise model, which is identifiable. However, both of these works focus on the SCM in the data space, while our approach focuses on learning the SCM in the latent space among the underlying latent variables in a weakly supervised setting. Other relevant work closely related to causal representation learning includes disentangled representations and independent component analysis [62,63,64,65,66].

## 3. Structural Causal Model

Following refs. Pearl [67], Bongers et al. [68], we describe the data-generating process (DGP) using the notion of structural causal models. A structural causal model (SCM) is a formal framework used to represent and analyze causal relationships among variables within a system. An SCM essentially consists of a set of random variables, and measurable functions between them specifying the underlying causal relationships of the DGP. We formally define SCMs as follows.

**Definition** **1**  (Structural Causal Model (SCM), Definition 2.1 by Bongers et al. [68])**.** *A structural causal model (SCM) is a tuple 〈L,J,E,Z,f,μ〉, where (i) L is a finite index set of endogenous variables; (ii) J is an index set of exogenous variables, which is disjoint with L; (iii) $E=\prod_{j\in J}E_{j}$ is the product of the domains of the exogenous variables, where each $E_{j}$ is a measurable space; (iv) $Z=\prod_{j\in L}Z_{j}$ is the product of the domains of the endogenous variables, where each $Z_{j}$ is a measurable space; (v) f:Z×E→Z is a measurable function that specifies the causal mechanism; and (vi) $\mu =\prod_{j\in J}\mu_{j}$ is a product measure, where $\mu_{j}$ is a probability measure on $E_{j}$ for each j∈J.*

In the definition above, the functional relationships between variables are expressed in terms of a function *f*. This feature allows us to model the cause–effect relationships of the data-generating process (DGP) using *structural equations*. Structural equations are mathematical representations used to describe causal relationships among variables in a system. They express how one or more variables causally influence others within a causal graphical model. For a given SCM as above, a structural equation specifies an endogenous random variable $z_{l}$ via a measurable function of the form $z_{l}=f_{l}\left(z,e\right)$ where z∈Z,e∈E. This function essentially captures the deterministic relationships specified by *f* as in Definition 1. A *parent*i∈L∪J of *l* is any index for which there is no measurable function $k:\prod_{j\in L\setminus \{i\}}Z_{j}\times E\to Z_{l}$ with $f_{l}=k$ almost surely. Intuitively, each endogenous variable $z_{l}$ is specified by its parents together with the exogenous variables via the structural equations.

**Figure 1:**
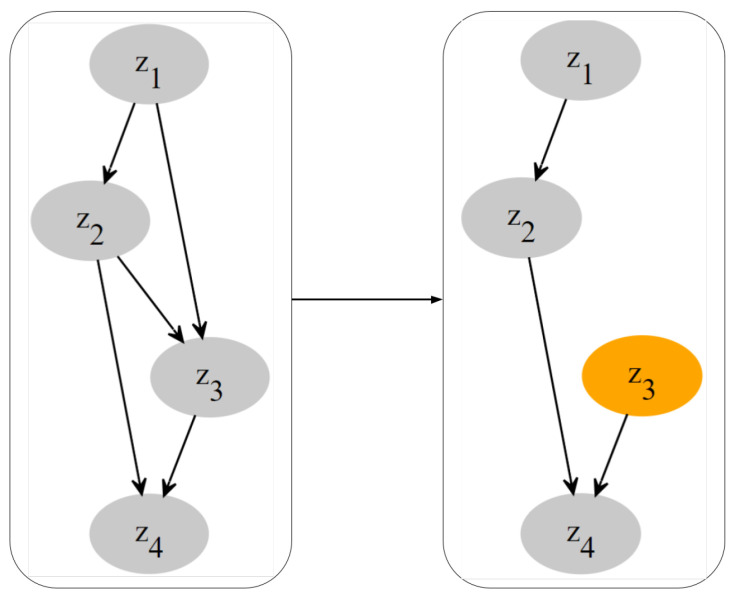
A causal graph before and after an intervention. Applying a perfect intervention on $z_{3}$ eliminates the dependencies between this node and its parents in the causal graph.

A structural equation model as in Definition 1 can be conveniently described with the *causal graph*, a directed graph of the form G=(V,E). The nodes of the causal graph consist of the entire set of indices for the endogenous variables, and the edges are specified by the structural equations, i.e., {j→l}∈E if and only if *j* is a parent of *l*. Note that the variables in the set $\mathrm{pa}(z_{l})$ are indexed by the parent nodes of *l* in the corresponding graph G. An example of a causal diagram is given in Figure 1(left).

**Solution Functions.** An alternative way of defining SCMs replaces causal mechanisms with solution functions h:E→Z which maps exogenous noise variables to endogenous causal variables, i.e., $z_{i}=h_{i}\left(e\right),e\in E$, and is defined by successively applying the causal mechanisms *f*. Solution functions contain the same information as causal mechanisms and they can be derived from each other. We utilize this formulation in our framework.

**Interventions.** A very important aspect of SCMs is that they allow us to reason about cause–effect relationships using *interventions*. Interventions refer to deliberate changes or manipulations made to one or more variables within the model to study their causal effects on other variables. In this paper, we specifically consider perfect interventions [67]. For a given SCM as in Definition 1, consider a variable $W:=\prod_{j\in L^{\prime }}Z_{j}$ for a set $L^{\prime }\subseteq L$, and let $w:=\prod_{j\in L^{\prime }}w_{j}$ be a point of its domain. The perfect intervention W←w amounts to replacing the structural equations $z_{j}=f_{j}\left(z,e\right)$ with the constant functions $z_{j}\equiv w_{j}$ for all $j\in L^{\prime }$. We denote with z∣do(w) the variables *z* after performing the interventions. This procedure defines a new probability distribution $p_{z}\left(z|do\left(w\right)\right)$, which we refer to as interventional distribution. This distribution entails the following information: If we apply do(w), what will be the value of *z*? We extend this definition by defining *I* as the set of interventions entailed by *w*, and we utilize this formulation in our framework. An example of a causal graph and a single perfect intervention is depicted in Figure 1.

**Equivalence of SCMs.** We now define the concept of equivalence between structural causal models. Two SCMs are structurally equivalent if their respective sets of structural equations and exogenous variables are equivalent. Formally, the notion of equivalence is defined as follows.

**Definition 2.**  *Consider two SCMs 〈L,J,E,Z,f,μ〉 and $\langle L^{\prime },J^{\prime },E^{\prime },Z^{\prime },f^{\prime },\mu^{\prime }\rangle$. Consider their respective causal graphs G and $G^{\prime }$. An isomorphism between the two SCMs consists of the following:*

*(A)*  *A graph isomorphism $\sigma :G\to G^{\prime }$; (A graph isomorphism $\sigma :G\to G^{\prime }$ is a bijective map from the vertices of G to the vertices of $G^{\prime }$, such that there exist an edge σ(i)→σ(j) in $G^{\prime }$ iff. there exist an edge i→j in G.)*
*(B)*  *Measure-preserving (A measure-preserving function l:A→B ensures that the probability distribution in the domain space A remains the same when mapped to the co-domain space B through the function l.) invertible functions $l_{j}:Z_{j}\to Z_{\sigma (j)}^{\prime }$ such that the function $l\left(z\right)\prod_{j\in L}l_{j}\left(z_{j}\right)$ yields $f^{\prime }\left(l\left(z\right),e\right)=l\left(f\left(z,e\right)\right)$ for all z∈Z,e∈E.*

*We say that two SCMs are equivalent if their domains are identical and such an isomorphism exists between them.*

Definition 2 ensures that the causal mechanisms of equivalent SCMs are essentially identical. The functions $l_{j}$ in Definition 2 reparameterize the random variables in both models such that the structural equations and causal relationships are preserved.

## 4. Diffusion Models

### 4.1. Overview

The fundamental concept behind diffusion-based generative models is to learn to generate data by inverting the diffusion process. Diffusion models comprise two processes: a forward process and a backward process. The forward process gradually adds noise to data and maps data to (almost) pure noise. The backward process, on the other hand, is used to go from a noise sample back to the original data space.

The forward process is defined by a stochastic differential equation (SDE) across a continuous time domain t∈[0,1], aiming to transform the data distribution to a known prior distribution, typically a standard multivariate Gaussian. Given $x_{0}$ sampled from a data distribution $p(x_{0})$, the forward process constructs a trajectory ${\left(x_{t}\right)}_{t\in [0,1]}$ across the time domain. We utilize the Variance Exploding SDE [53] for the forward process, which is defined as:

$$dx=f\left(x,t\right)+g\left(t\right)dw:=\sqrt{\frac{d[\sigma^{2}\left(t\right)]}{dt}}dw,$$

where *w* is the standard Wiener process, and $\sigma^{2}\left(t\right)$ is the noise variance of the diffusion process at time *t*. The backward process is also formulated as an SDE in the following manner:

$$dx=\left[f\left(x,t\right)-g^{2}\left(t\right)\nabla_{x}\log p_{t}\left(x\right)\right]dt+g\left(t\right)d\bar{w}\;,$$

where $\bar{w}$ is the standard Wiener process in reverse time.

**Score matching.** To use this backward process, the score function $\nabla_{x}\log p_{t}\left(x\right)$ is required. It is usually approximated by a neural score function $s_{\theta }\left(\cdot \right)$ which can be trained by Explicit Score Matching [69] defined as:

$$\begin{array}{c} L\left(\theta \right)=E_{t}[\lambda \left(t\right)E_{p(x_{t})}[\left|\right|s_{\theta }\left(x_{t},t\right)-\nabla_{x_{t}}\log p_{t}\left(x_{t}\right){\left|\right|}^{2}]], \end{array}$$

where λ(t) is a positive weighting function. However, the ground-truth score function $\nabla_{x}\log p_{t}\left(x\right)$ is generally not known. Vincent [70] addresses this issue by proposing Denoising Score Matching. The approximate score function is then learned by minimizing the loss function:

$$\begin{array}{c} L\left(\theta \right)=[\lambda \left(t\right)E_{x_{0}}E_{p(x_{t}|x_{0})}\left[\left|\right|s_{\theta }\left(x_{t},t\right)-\nabla_{x_{t}}\log p_{t}\left(x_{t}|x_{0}\right){\left|\right|}^{2}\right]], \end{array}$$

where the conditional distribution of $x_{t}$ given $x_{0}$ is $p_{t}\left(x_{t}|x_{0}\right)=N\left(x_{t};x_{0},\left[\sigma^{2}\left(t\right)-\sigma^{2}\left(0\right)\right]I\right)$. This objective function originates from the Evidence Lower Bound (ELBO) of the data distribution, and it has been shown that with a specific weighting function, this objective function becomes exactly a term in the ELBO [53]. For more details, see Appendix B.

### 4.2. Diffusion-Based Representations

**Conditional Score Matching.** We can modify Denoising Score Matching so that the score function receives additional information through an external trainable module. This results in a conditional diffusion model which allows to perform representation learning while training the score function. Abstreiter et al. [43] proposes conditional Denoising Score Matching defined as:

(1) $$\begin{array}{c} L\left(\theta ,\phi \right)=E_{t}[\lambda \left(t\right)E_{x_{0}}E_{p(x_{t}|x_{0})}\left[\left|\right|s_{\theta }\left(x_{t},E_{\phi }\left(x_{0}\right),t\right)-\nabla_{x_{t}}\log p_{t}\left(x_{t}|x_{0}\right){\left|\right|}^{2}\right]], \end{array}$$

where the score function is conditioned on a module $E_{\phi }\left(x_{0}\right)$ which provides additional information about the data to the diffusion model through a learned encoder with parameters ϕ. In fact, the encoder learns to extract necessary information from $x_{0}$ in a reduced-dimensional space that helps recover $x_{0}$ by denoising $x_{t}$. Abstreiter et al. [43] also presents an alternative objective where the encoder is a function of time. Formally, the new objective is

(2) $$\begin{array}{c} L\left(\theta ,\phi \right)=E_{t}[\lambda \left(t\right)E_{x_{0}}E_{p(x_{t}|x_{0})}\left[\left|\right|s_{\theta }\left(x_{t},E_{\phi }\left(x_{0},t\right),t\right)-\nabla_{x_{t}}\log p_{t}\left(x_{t}|x_{0}\right){\left|\right|}^{2}\right]], \end{array}$$

With this objective, the encoder learns a representation trajectory of $x_{0}$ instead of a single representation. Training this system has the potential to minimize the objective to zero, motivating the encoder $E_{\phi }\left(.\right)$ to learn meaningful, distinct representations at different timesteps [43,44].

**Comparison with Other Generative Models.** The key difference between the other generative models and diffusion-based representations is that other generative models are only concerned with one finite code and all the information is encoded into this single code, while in the latter, different levels of information are encoded along an infinite-dimensional code, i.e., the encoder is conditioned on time *t* and produces a trajectory-based representation ${\left(E_{\phi }\left(x_{0},t\right)\right)}_{t\in [0,1]}$. Within this representation, various points along the trajectory contain different levels of information as highlighted by Mittal et al. [44]. In this work, we first explore a time-independent single code, where we employ Equation (1) and show that with a certain weighting function, this objective function will become the ELBO. Then, we apply the same experiments with infinite-dimensional latent code (Equation (2)) and study the benefits and implications of these formulations for causal representation learning.

## 5. Problem Formulation

We consider a system that is described by an unknown underlying SCM on the latent causal variable *z*, where we have access to low-level data pairs $\left(x_{0},{\tilde{x}}_{0}\right)∼p\left(x_{0},{\tilde{x}}_{0}\right)$ representing the system before and after a random, unknown, and atomic intervention. We consider the assumptions and the data-generation process that will be described in Section 5.1. Our objective is to learn an SCM that accurately represents the true underlying SCM associated with the given data, up to a permutation and elementwise reparameterization of causal variables and solution functions. To this end, we train an SCM by maximizing the likelihood of data. With sufficient data and perfect optimization, we can find the SCM that is equivalent to the ground-truth SCM.

### 5.1. Weakly Supervised Framework

We build our weakly supervised framework on the assumptions and identifiability conditions established by Brehmer et al. [14]. We try to learn the underlying SCM over unknown latent causal variables *z* of a system in which low-level information $x_{0}\in X$ generated directly from *z* through an unknown function g:Z→X is available. Following Brehmer et al. [14], Locatello et al. [26], we consider a dataset that consists of paired datapoints $\left(x_{0},{\tilde{x}}_{0}\right)$, generated as follows:

$$\begin{array}{cc} \; & e∼p_{e}\left(e\right),\;I∼p_{I}\left(I\right),\;z=h\left(e\right),\;x_{0}=g\left(z\right)\\ & \tilde{e}∼p_{e}\left(e|do\left(e^{\prime }\right)\right)\;\mathrm{with}\;e^{\prime }∼p_{e_{I}}\left(e^{\prime }\right),\;\tilde{z}={\tilde{h}}_{I}\left(\tilde{e}\right),\;{\tilde{x}}_{0}=g\left(\tilde{z}\right) \end{array}$$

where *e* and $\tilde{e}$ are the exogenous noise variables of the underlying SCM, h(·) and ${\tilde{h}}_{I}\left(\cdot \right)$ are the solution functions before and after a single perfect intervention *I*, and $p_{I}\left(\cdot \right)$ is a prior on all possible values of atomic interventions such that $p_{e_{I}}\left(e^{\prime }\right)>0$ for every possible atomic intervention. In this setting, $p_{e}\left(e|do\left(e^{\prime }\right)\right)$ is defined such that the noise variable remains the same and changes only for the element that is intervened upon, i.e., ${\tilde{e}}_{I}=e^{\prime }\neq e_{I},\;{\tilde{e}}_{\setminus I}=e_{\setminus I}$. Since the intervention is perfect, the solution function will also change in a way that only for the intervened variable is the dependency between the latent causal variable $z_{I}$ and its parents removed. For the complete list of assumptions, see Appendix A.

It is proven that under this weakly supervised setting, it is possible to identify the latent causal variables and solution functions up to a permutation and elementwise reparameterization of the variables. For the proof of the identifiability of the described system, we refer to Brehmer et al. [14].

### 5.2. Non-Identifiability from Observational Data

In this section, we show that interventions are necessary for identifiability in this setting. In fact, note that Definition 2 implies that the distributions of two equivalent SCMs are the same, up to a measure-preserving invertible function. However, two SCMs may entail the same observational distribution on the generated data, even if their respective causal mechanisms are not equivalent. This is best illustrated with an example. Consider two datasets $\left\{X_{1},Y_{1}\right\}$ and $\left\{X_{2},Y_{2}\right\}$. The respective DGPs are:

$$X_{1},Y_{1}∼N\left(0,\Sigma \right)\;\mathrm{and}\;\left\{\begin{array}{c} X_{2}∼N\left(0,1\right)\\ \;Y_{2}∼X_{2} \end{array}\right.$$

where the covariance matrix Σ is defined as

$$\Sigma =\left[\begin{array}{cc} 1 & 1\\ 1 & 1 \end{array}\right].$$

Note that both datasets $\left\{X_{1},Y_{1}\right\}$ and $\left\{X_{2},Y_{2}\right\}$ entail the same observational distribution. However, these datasets have different causal mechanisms. In particular, their respective causal diagrams are not isomorphic. Hence, by this, we see that the same observational distribution may entail different causal diagrams. This means that the causal dynamics of an SCM cannot be inferred from the distribution of a given observational dataset, i.e., SCMs are unidentifiable from observational data.

### 5.3. Limitations

While our goal is to execute a robust and informative study to address the selected research question, it is important to acknowledge inherent limitations related to data, model assumptions, and evaluations. First, our evaluation is limited to synthetic datasets in a single modality. Furthermore, we consider the weakly supervised data-generation process and assumptions for the identifiability of the underlying model, which may limit the practical application of our work in systems where the assumptions do not hold. Finally, the representation learning process relies on an encoder, which acts as an information channel, regulating the amount of input information transmitted to the score function during each step of the diffusion process. It is important to note that in certain scenarios, the encoder may not be essential to the diffusion process and could potentially result in collapsing behavior. However, it is important to emphasize that our work is a preliminary step towards utilizing diffusion models for causal representation learning and lays the foundation for significant further research in this area.

## 6. The DCRL Framework

**Figure 2:**
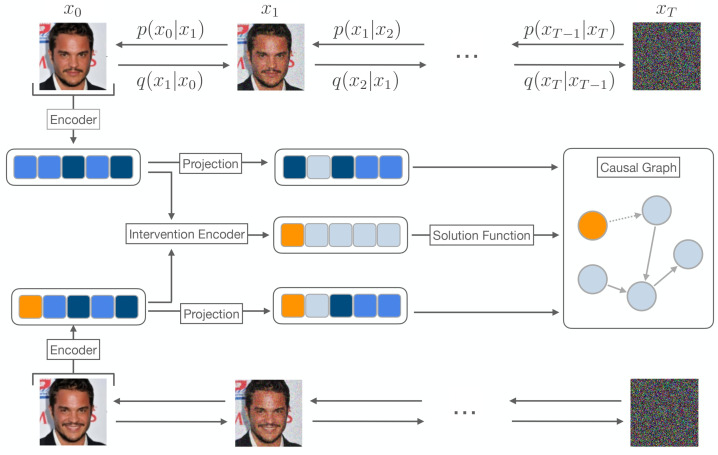
Overview of our framework. Here, we have a paired image of a face before and after an intervention (the smile). The paired image is mapped to latent variables by a stochastic encoder. The intervention target is determined by applying the intervention encoder to these latent variables. To maintain the weakly supervised structure, the latent variables are projected into a new pair and then serve as the conditioning module for a conditional diffusion model. The projected latent variables are in fact diffusion-based representations of the input pair. Finally, they are utilized in neural solution functions together with the intervention target to obtain the latent causal variables.

Figure 2 provides a visual representation of the framework’s architecture. In this study, we utilize a conditional diffusion model and apply it to the input data ($x_{0}$, ${\tilde{x}}_{0}$), where $x_{0},{\tilde{x}}_{0}\in R^{3\times W\times H}$ and *W* and *H* are the width and height of the input, respectively. We denote ${\left(x_{t}\right)}_{t\in [0,1]}$ as the diffusion trajectory across the time domain with $x_{0}$ as the input data. The conditioning module is defined as the encoding module, generating high-level diffusion-based representations $\left(e,\tilde{e}\right)$ for each low-level data pair, where $e,\tilde{e}\in R^{d}$ and *d* is the number of latent causal variables assumed to be known. We empirically show that these latent variables contain equivalent information as in noise variables of the underlying SCM and can be used interchangeably. Then, we infer the intervention target I∈{0,1,...,d−1} for each data pair by an intervention module and use neural solution functions on top of the latent variables $\left(e,\tilde{e}\right)$ and the intervention target *I* to obtain the underlying latent causal variables $z,\tilde{z}\in R^{d}$. We base our framework on the Implicit Latent Causal Model (ILCM) introduced by Brehmer et al. [14] and describe each part of our framework in the next paragraphs.

### 6.1. Conditional Diffusion Model

Based on the formulation described in Section 4, we use a conditional diffusion model. A stochastic encoder $q(e|x_{0})$ serves as the conditioning module, mapping low-level data space to high-level latent space. When employing a finite code where the stochastic encoder is independent of time, *e* is a single vector of size *d*. In this case, the framework learns a single SCM. Alternatively, in the case of using infinite-dimensional latent code, the stochastic encoder generates ${\left(e_{t}\right)}_{t\in [0,1]}$ which is a trajectory-based representation across time. At each timestep *t*, $e_{t}\in R^{d}$ represents a single point of the trajectory. In this scenario, the framework learns an SCM at each timestep. In the following paragraphs, for the sake of simplicity, we use the single-code formulation.

### 6.2. The Encoding and the Intervention Module

The encoding module consists of two main parts: the *stochastic encoder* and the *projection module*. The stochastic encoder $q(e|x_{0})$ maps data pairs ($x_{0}$, ${\tilde{x}}_{0}$) to pre-projection latent variables (*e*, $\tilde{e}$). The encoded inputs are then utilized in the intervention module $q(I|x_{0},{\tilde{x}}_{0})$ to infer the intervention target *I* for the data pair ($x_{0}$, ${\tilde{x}}_{0}$). Based on our data generation process in Section 5.1, the encoded inputs have the property that only for the elements that are intervened upon do we have $e_{i}\neq {\tilde{e}}_{i},i\in I$, and the rest will remain the same. Based on this property, in order to infer interventions, we employ an intervention module $q(I|x,\tilde{x})$ which is defined heuristically as

$$q\left(i\in I|x_{0},{\tilde{x}}_{0}\right)=\frac{1}{Z}\left(\alpha +\beta \right|\mu_{e}{\left(x_{0}\right)}_{i}-\mu_{e}{\left({\tilde{x}}_{0}\right)}_{i}\left|+\gamma \right|\mu_{e}{\left(x_{0}\right)}_{i}-\mu_{e}{\left({\tilde{x}}_{0}\right)}_{i}{|}^{2})$$

where $\mu_{e}\left(x_{0}\right)$ is the mean of the stochastic encoder $q(e|x_{0})$; α, β, and γ are learnable parameters; and *Z* is a normalization constant. This simple heuristic function ensures that a variable has a higher chance to be selected as the intervened variable if it undergoes more significant changes in response to the intervention. Once the intervention is inferred from the pre-projection latent variables, we apply the projection module. Similar to Brehmer et al. [14], the projection module is dependent on the inferred intervention target *I* and projects the encoded input $\left(e,\tilde{e}\right)$ to new latent variables in a way that for the components $e_{i}$ that are not intervened upon i∉I, the pre-intervention and post-intervention latent components will be equal $e_{i}={\tilde{e}}_{i}$. This prevents the framework from deviating from the weakly supervised structure.

We write the combination of the encoder and the projection module as $q(e,\tilde{e}|x_{0},{\tilde{x}}_{0},I)$, and refer to it as the *encoding module*. By this definition, the encoding module $q(e,\tilde{e}|x_{0},{\tilde{x}}_{0},I)$ maps the input ($x_{0}$, ${\tilde{x}}_{0}$) to latent variables (*e*, $\tilde{e}$) and the intervention module infers the intervention *I* based on pre-projection latent variables.

### 6.3. Prior

Given the intervention target *I* and latent variables (*e*, $\tilde{e}$), we define the prior $p(e,\tilde{e},I)$ as $p\left(e,\tilde{e},I\right)=p\left(I\right)p\left(e\right)p\left(\tilde{e}|e,I\right)$. The objective of the prior distribution is to implicitly capture the causal structure and causal mechanisms within the system. Specifically, p(I) and p(e) denote the prior distributions over intervention targets and latent variables, respectively, and are configured as uniform categorical with each latent variable as a category, and standard Gaussian distributions, respectively. According to our data generation process, when an intervention is applied, only the elements in the latent variables that are intervened upon are altered; the other elements remain unchanged and independent of each other. Consequently, we can define $p(\tilde{e}|e,I)$ as follows:

$$p\left(\tilde{e}|e,I\right)=\prod_{i\notin I}\delta \left({\tilde{e}}_{i}-e_{i}\right)\prod_{i\in I}p\left({\tilde{e}}_{i}|e\right)$$

In this equation, δ(.) is the Dirac delta function that fulfills this property for non-intervened latent variables.

### 6.4. Neural Solution Functions

In order to encode the information about the intervened variables, we incorporate a conditional normalizing flow $p({\tilde{e}}_{i}|e)$ defined as

$$p\left({\tilde{e}}_{i}|e\right)=\tilde{p}\left(h_{i}\left({\tilde{e}}_{i};e_{i}\right)\right)|\frac{\partial h_{i}\left({\tilde{e}}_{i};e_{i}\right)}{\partial {\tilde{e}}_{i}}|$$

where h(.) are the solution functions of the SCM. They are defined as invertible affine transformations with parameters learned with neural networks. Therefore, by learning solution functions, i.e., learning to transform *e* to *z*, we implicitly model the causal graph into the framework and obtain the latent causal variables. For more details about the implementation, see Appendix C.

### 6.5. The Evidence Lower Bound for DCRL

We calculate the Evidence Lower Bound (ELBO) for the proposed model for the framework described in the previous section. In the case of having single-point representations in which the noise variable *e* is independent of time, the ELBO becomes:

$$\begin{array}{cc} L_{model} & =E_{p(x_{0},{\tilde{x}}_{0})}E_{q(I|x_{0},{\tilde{x}}_{0})}E_{q(e,\tilde{e}|x_{0},{\tilde{x}}_{0},I)}E_{t∼U(0,1)}E_{q(x_{t}|x_{0})}E_{q({\tilde{x}}_{t}|{\tilde{x}}_{0})}[\lambda \left(t\right)\left|\right|s_{\theta }\left(x_{t},e,t\right)\\ & -\nabla_{x_{t}}\log p\left(x_{t}|x_{0}\right){\left|\right|}_{2}^{2}+\lambda \left(t\right)\left|\right|s_{\theta }\left({\tilde{x}}_{t},\tilde{e},t\right)-\nabla_{{\tilde{x}}_{t}}\log p\left({\tilde{x}}_{t}|{\tilde{x}}_{0}\right){\left|\right|}_{2}^{2}+\beta [\log p\left(I\right)+\log p\left(e\right)\\ \; & +\log p\left(\tilde{e}|e,I\right)-\log q\left(I|x_{0},{\tilde{x}}_{0}\right)-\log q\left(e,\tilde{e}|x_{0},{\tilde{x}}_{0},I\right)]], \end{array}$$

where λ(t) is a positive weighting function, and β=1. We train the model by minimizing a reweighted loss function reminiscent of β-VAEs, setting β to 0 and increasing it to 1 during training.

In the case of using infinite-dimensional representations (Equation (2)), the objective function becomes:

(3) $$\begin{array}{cc} L_{model} & =E_{p(x_{0},{\tilde{x}}_{0})}E_{q(I|x_{0},{\tilde{x}}_{0})}E_{t∼U(0,1)}E_{q(e_{t},\tilde{e_{t}}|x_{0},{\tilde{x}}_{0},I)}E_{q(x_{t}|x_{0})}E_{q({\tilde{x}}_{t}|{\tilde{x}}_{0})}[\lambda \left(t\right)\left|\right|s_{\theta }\left(x_{t},e_{t},t\right)\\ \; & -\nabla_{x_{t}}\log p\left(x_{t}|x_{0}\right){\left|\right|}_{2}^{2}+\lambda \left(t\right)\left|\right|s_{\theta }\left({\tilde{x}}_{t},\tilde{e_{t}},t\right)-\nabla_{{\tilde{x}}_{t}}\log p\left({\tilde{x}}_{t}|{\tilde{x}}_{0}\right){\left|\right|}_{2}^{2}+\beta [\log p\left(I\right)+\log p\left(e_{t}\right)\\ \; & +\log p\left({\tilde{e}}_{t}|e_{t},I\right)-\log q\left(I|x_{0},{\tilde{x}}_{0}\right)-\log q\left(e_{t},{\tilde{e}}_{t}|x_{0},{\tilde{x}}_{0},I\right)]], \end{array}$$

where ${\left(e_{t}\right)}_{t\in [0,1]}$ is the trajectory-based representation and $e_{t}\in R^{d}$ is the single point of the trajectory at time *t*. For a complete derivation of the ELBO, see Appendix B.

To prevent a collapse of the latent space to a lower-dimensional subspace, we add the negative entropy of the batch-aggregate intervention posterior as a regularization term to the loss function:

$$L_{entropy}=E_{batches}\left[-\sum_{I}q_{I}^{batch}\left(I\right)\log q_{I}^{batch}\left(I\right)\right]$$

where $E_{batches}\left[\;\cdot \;\right]$ is the expected value over all the batches of data, and $q_{I}^{batch}\left(I\right)$ is defined as

$$q_{I}^{batch}\left(I\right)=E_{x_{0},{\tilde{x}}_{0}\in batch}\left[q\left(I|x_{0},{\tilde{x}}_{0}\right)\right]$$

After the training, the framework contains information about the underlying causal structure and latent causal variables, and it can be used in different downstream tasks.

## 7. Experiments

Here, we analyze the performance of the proposed model, DCRL, on synthetic data. We employ DCRL for the task of causal discovery. After training DCRL, we use the framework to obtain causal variables $\left(z,\tilde{z}\right)$ for the test set, and apply ENCO [71], a continuous optimization structure learning method that leverages observational and interventional data, on the obtained samples to infer the underlying causal graph. Furthermore, we evaluate the learned causal variables with the DCI framework [72].

**Data Generation.** In order to generate latent causal variables, we adopt random graphs, where each edge in a fixed topological order is sampled from a Bernoulli distribution with a parameter that is equal to 0.5. We consider the SCM to be linear Gaussian and we sample the weights from a multivariate normal distribution with zero mean and unit variance. We make sure the weights are not close to zero to avoid violation of the faithfulness assumption. We introduce additive Gaussian noise with equal variances across all nodes, with its variance set to 0.1. Latent causal variables are then sampled using ancestral sampling, and we generate $10^{5}$ training samples, $10^{4}$ validation samples, and $10^{4}$ test samples. Finally, to generate input data $x_{0}$, we apply a random linear projection on the obtained latent variables. We keep the dimension of $x_{0}$ fixed to 16. We utilize an SCM with 5, 10, and 15 variables. To enhance the robustness of the results, we generate data for 4 different seeds and repeat our experiments for each seed.

**Baselines.** We consider ILCM [14] as our main baseline. To the best of our knowledge, there are no other methods that consider the same weakly supervised assumptions, and adapting other methods to our assumptions either substantially changes the method or is infeasible. We also evaluate the outcomes against a variation of disentanglement VAE proposed by [26] tailored for weakly supervised settings. This model, referred to as d-VAE, models the weakly supervised process but assumes unconnected variation factors instead of a causal relationship among variables. Similarly, we apply ENCO on top of both to obtain the learned graph.

**Metrics.** We assess the performance of models with the following metrics:

- The *Structural Hamming Distance (SHD)* is a metric used to quantify the dissimilarity between two directed acyclic graphs (DAGs) by measuring the minimum number of edge additions, deletions, and reversals required to transform one graph into another. It is calculated by summing up the absolute differences between the entries of adjacency matrices of two graphs.
- The *DCI Disentanglement Score* is a metric used to evaluate the disentanglement quality of a generative model and takes values between 0 and 1. Disentanglement refers to the extent to which the model learns to predict the underlying factors of variation in the data in a way that each predicted variable captures at most one underlying factor. If a predicted factor is important to predict a single underlying factor, the score will be 1, and if a predicted factor is equally important to predict all the underlying factors, the score will be 0 [72].
- The *DCI Completeness Score* measures how well each underlying factor of variation is captured by a single predicted latent variable and has a value between 0 and 1. If a single variable contributes to one underlying factor, the score will be 1, and if all variables equally contribute to the prediction of a single factor, the score will be 0 [72].

### 7.1. Single-Point Representations

**Figure 3:**
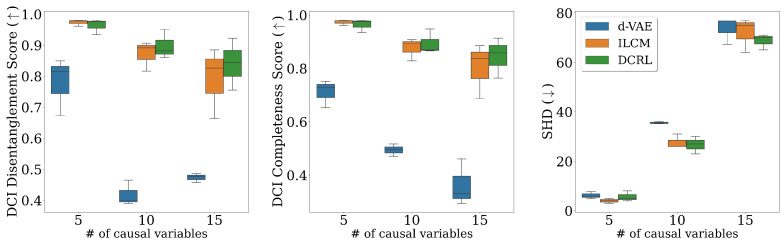
Comparison of models on different metrics when using single-point representations. Our approach outperforms or competes favorably with the baseline methods on all metrics. Particularly in higher dimensions, our method excels by capturing additional information about the causal variables and the underlying causal structure.

Utilizing single-point representations where $e\in R^{d}$ and is independent of time, our method demonstrates superior or competitive performance compared to the baselines as indicated by the metrics shown in Figure 3. The d-VAE performs poorly across all metrics primarily because it assumes independent rather than causal relationships among variables. In scenarios involving 5 and 10 causal variables, ILCM shows comparable performance to DCRL, suggesting that a standard VAE can sufficiently capture essential information about causal factors. However, in higher dimensions, our method excels by capturing more detailed information about causal variables and their underlying structure. Our findings indicate that diffusion-based representations are more beneficial in higher dimensions, providing more accurate information about the underlying causal variables compared to other baseline methods.

### 7.2. Infinite-Dimensional Representations

**Figure 4:**
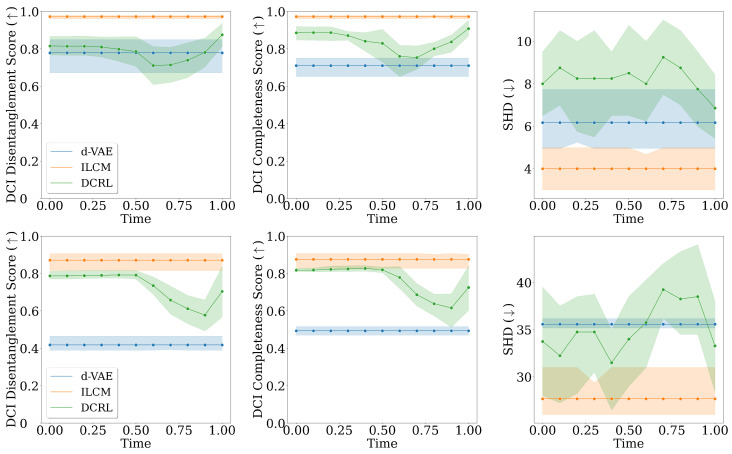

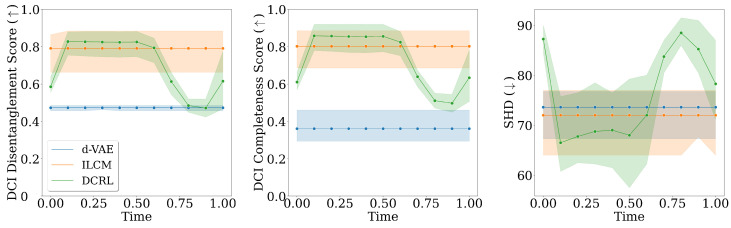
Comparison of models on different metrics when using infinite-dimensional representations. From top to bottom, each row corresponds to experiments with 5, 10, and 15 causal variables, respectively. We sample points from the trajectory at intervals of 0.1, creating a total of 11 specific timesteps. Typically, representations in the middle of the trajectory carry the most information, often matching or surpassing the baseline performance. As we move further in time, representations seem to lose some information, but they improve as they approach the end of the trajectory. Furthermore, the framework performs worse or on par with baselines in lower dimensions but generally outperforms them in higher dimensions.

In these experiments, we utilize the infinite-dimensional representations approach to develop trajectory-based representations for each input $x_{0}$, denoted as ${\left(e_{t}\right)}_{t\in [0,1]}$. In order to perform inference, we sample points from this trajectory at intervals of 0.1 resulting in 11 specific timesteps. The outcomes are depicted in Figure 4. Generally, representations in the middle of the trajectory contain the most information and are comparable to or even outperform the baselines. Going further in time, representations appear to lose information but improve as they move towards the end of the trajectory. This phenomenon arises because during training, as we are further in time, the noise in the diffusion model is fairly high and the conditioning module compensates for that by providing the necessary information for the diffusion model to learn the score function.

## 8. Conclusions

Identifying the underlying causal variables and mechanisms of a system solely from observational data is considered impossible without additional assumptions. In this project, we use weak supervision as an inductive bias and study whether the information encoded in the latent code of diffusion-based representations contains useful knowledge of causal variables and the underlying causal graph.

This study represents an initial exploration of applying diffusion models to causal representation learning, highlighting the need for further research and extensions in this area. Our method relies on an external encoder to provide necessary information for the diffusion model to learn the score function. Future work could focus on integrating more efficient ways of acquiring representations from diffusion models without external dependencies or conditioning. Additionally, extending the weakly supervised framework to higher dimensions and other modalities, such as video or multi-view data, is another potential direction. Applying the proposed method to domains such as experimental design, reinforcement learning, and robotics—where the independent actions can be considered interventions and the system’s state before and after an action is observable—presents another promising avenue for research. Finally, extending the framework to other settings, such as dynamical systems, where the infinite-dimensional latent code corresponds to the system’s state at different timesteps, is another interesting potential direction.

## Data Availability

The original contributions presented in the study are included in the article, further inquiries can be directed to the corresponding author.

## Appendix A. Identifiability Conditions

We give all the assumptions used by [14] for the SCM to be identifiable up to a permutation and elementwise reparameterization of the causal variables. These assumptions are as follows:

- **Causal Sufficiency** (see Definition 6.2.2 by Pearl [67]). All the causal variables are measurable, and the noise variables are mutually independent.
- **Faithfulness** (see Definition 2.4.1 by Pearl [67]). All the independencies of data distribution are encoded in the graph.
- **Acyclicity.** The ground-truth graph is acyclic.
- **Diffeomorphic Causal Mechanisms** (see Brehmer et al. [14]). We require causal mechanisms and, therefore, solution functions h(·) to be diffeomorphic, that is, for any possible input value of the causal mechanisms f(·), *f* is invertible, differentiable, and its inverse is differentiable.
- **Observability of All Interventions** (see Brehmer et al. [14]). The intervention distribution $p_{I}\left(\cdot \right)$ has support for any atomic intervention, i.e., $p_{I}\left(z_{j}\right)>0,\;\forall j\in L$. In other words, the dataset contains data pairs generated from interventions on any causal variable.
- **Perfect Atomic Interventions** (see Section 3.2. by Pearl [67], and Brehmer et al. [14]). We assume that the intervention set *I* contains atomic interventions on causal variables in which the intervention is perfect, i.e., the intervened-upon mechanism is independent of any causal variable.

## Appendix B. Problem Formulation and ELBO Derivation

Here, we derive the ELBO for the proposed framework. To avoid confusion, in the notation, we separate the latent variables of the diffusion model and the input data. We denote them with *u* and $x_{0}$, respectively. Furthermore, for simplicity, we only derive the ELBO when using single-point representations independent of time, i.e., $e\in R^{d}$. The ELBO for the infinite-dimensional can be derived similarly. The ELBO for the framework is calculated as:

$$\begin{array}{cc} \log\; & p\left(x_{0},{\tilde{x}}_{0}\right)\geq E_{q(e,\tilde{e},u,\tilde{u},I|x_{0},{\tilde{x}}_{0})}\left[\log\frac{p(x_{0},{\tilde{x}}_{0},u,\tilde{u},e,\tilde{e},I)}{q(e,\tilde{e},I,u,\tilde{u}|x_{0},{\tilde{x}}_{0})}\right]\\ = & E_{q(e,\tilde{e},u,\tilde{u},I|x_{0},{\tilde{x}}_{0})}\left[\log\frac{p(I)}{q(I|x_{0},{\tilde{x}}_{0})}+\log\frac{p\left(e\right)p\left(\tilde{e}|e,I\right)}{q(e,\tilde{e}|x_{0},{\tilde{x}}_{0},I)}+\log\frac{p(x_{0},u|e)}{q(u|x_{0})}+\log\frac{p({\tilde{x}}_{0},\tilde{u}|\tilde{e})}{q(\tilde{u}|{\tilde{x}}_{0})}\right]\\ = & E_{q(I|x_{0},{\tilde{x}}_{0})}E_{q(e,\tilde{e}|x_{0},{\tilde{x}}_{0},I)}E_{q(u|x_{0})}E_{q(\tilde{u}|{\tilde{x}}_{0})}[[\log p\left(I\right)+\log p\left(e\right)+\log p\left(\tilde{e}|e,I\right)\\ - & \log q\left(I|x_{0},{\tilde{x}}_{0}\right)-\log q\left(e,\tilde{e}|x_{0},{\tilde{x}}_{0},I\right)]+\left[\log\frac{p(x_{0},u|e)}{q(u|x_{0})}+\log\frac{p({\tilde{x}}_{0},\tilde{u}|\tilde{e})}{q(\tilde{u}|{\tilde{x}}_{0})}\right]] \end{array}$$

The terms in the first bracket correspond to the intervention encoder and the noise encoding module, respectively, and the terms in the second bracket correspond to the diffusion model conditioned on pre- and post-intervention noise encodings.

Song et al. [53] shows that the discretization of SDE formulations of the diffusion model is equivalent to discrete-time diffusion models. Therefore, for simplicity, we derive the ELBO for discrete-time diffusion models. Following [73], for a discrete-time diffusion model where t∈]1,T], we have

(A1) $$\begin{array}{cc} E_{q(I|x_{0},{\tilde{x}}_{0})} & E_{q(e,\tilde{e}|x_{0},{\tilde{x}}_{0},I)}E_{q(u|x_{0})}E_{q(\tilde{u}|{\tilde{x}}_{0})}\left[\log\frac{p(x_{0},u|e)}{q(u|x_{0})}\right]=\\ \; & \;\;E_{q(e,\tilde{e}|x_{0},{\tilde{x}}_{0},I)}[E_{q(u_{1}|x_{0})}\left[\log p\left(x_{0}|u_{1}\right)\right]-D_{KL}(q\left(u_{T}|x_{0}\right)||p\left(u_{T}\right))\\ \; & \;\;-\sum_{t=2}^{T}E_{q(u_{t}|x_{0})}[D_{KL}(q\left(u_{t-1}|u_{t},x_{0},e\right)||p\left(u_{t-1}|u_{t},e\right)]] \end{array}$$

where we have the following:

- $E_{q(u_{1}|x_{0})}\left[\log p\left(x_{0}|u_{1}\right)\right]$ is the reconstruction term, and it can be defined in a way that it is constant so it can be ignored during training.
- $D_{KL}(q\left(u_{T}|x_{0}\right)||p\left(u_{T}\right))$ is the prior matching term and can similarly be defined in a way that it is constant.
- $E_{u_{t}|x_{0}}[D_{KL}(q\left(u_{t-1}|u_{t},x_{0},e\right)||p\left(u_{t-1}|u_{t},e\right)]$ is a denoising matching term. This term is the origin of different interpretations of the score-based diffusion models.

For the SDE formulation of the forward diffusion process, the denoising matching term becomes [53]

(A2) $$\lambda \left(t\right)||s_{\theta }\left(u_{t},e,t\right)-\nabla_{u_{t}}\log p\left(u_{t}|x_{0}\right){\left|\right|}_{2}^{2}.$$

The weight λ(t) of denoising matching terms is related to the diffusion coefficient of the forward SDE. For a Variance Exploding SDE, the weight is defined as $\lambda \left(t\right)=2\sigma^{2}\left(t\right)\log\left(\sigma_{max}/\sigma_{min}\right)$ with $\sigma \left(t\right)=\sigma_{min}\cdot {\left(\sigma_{max}/\sigma_{min}\right)}^{t}$. Therefore, by combining (A1) with (A2), the ELBO becomes

$$\begin{array}{cc} \log p(x_{0},{\tilde{x}}_{0}) & \geq E_{p(x_{0},{\tilde{x}}_{0})}E_{q(I|x_{0},{\tilde{x}}_{0})}E_{q(e,\tilde{e}|x_{0},{\tilde{x}}_{0},I)}E_{t∼U(0,1)}E_{q(u_{t}|x_{0})}E_{q({\tilde{u}}_{t}|{\tilde{x}}_{0})}\\ {}[ & \log p\left(I\right)+\log p\left(e\right)+\log p\left(\tilde{e}|e,I\right)-\log q\left(I|x_{0},{\tilde{x}}_{0}\right)-\log q\left(e,\tilde{e}|x_{0},{\tilde{x}}_{0},I\right)\\ + & \lambda \left(t\right)\left[\left|\right|s_{\theta }\left(u_{t},e,t\right)-\nabla_{u_{t}}\log p\left(u_{t}|x_{0}\right){\left|\right|}_{2}^{2}+\left|\right|s_{\theta }\left({\tilde{u}}_{t},\tilde{e},t\right)-\nabla_{{\tilde{u}}_{t}}\log p\left({\tilde{u}}_{t}|{\tilde{x}}_{0}\right){\left|\right|}_{2}^{2}\right]] \end{array}$$

For infinite-dimensional representations, we can derive the ELBO using a similar argument. In this case, the formula for the ELBO is

$$\begin{array}{cc} \log p(x_{0}, & {\tilde{x}}_{0})\geq E_{p(x_{0},{\tilde{x}}_{0})}E_{q(I|x_{0},{\tilde{x}}_{0})}E_{t∼U(0,1)}E_{q(e_{t},{\tilde{e}}_{t}|x_{0},{\tilde{x}}_{0},I)}E_{q(u_{t}|x_{0})}E_{q({\tilde{u}}_{t}|{\tilde{x}}_{0})}\\ {}[ & \log p\left(I\right)+\log p\left(e_{t}\right)+\log p\left({\tilde{e}}_{t}|e_{t},I\right)-\log q\left(I|x_{0},{\tilde{x}}_{0}\right)-\log q\left(e_{t},{\tilde{e}}_{t}|x_{0},{\tilde{x}}_{0},I\right)\\ + & \lambda \left(t\right)||s_{\theta }\left(u_{t},e_{t},t\right)-\nabla_{u_{t}}\log p\left(u_{t}|x_{0}\right){\left|\right|}_{2}^{2}+\lambda \left(t\right)\left|\right|s_{\theta }\left({\tilde{u}}_{t},{\tilde{e}}_{t},t\right)-\nabla_{{\tilde{u}}_{t}}\log p\left({\tilde{u}}_{t}|{\tilde{x}}_{0}\right){\left|\right|}_{2}^{2}] \end{array}$$

## Appendix C. Implementation Details

### Appendix C.1. Training

For the training, we follow the four-phase training of Brehmer et al. [14] but consider only the first three phases. In summary, we consider the following steps:

(1) We begin by training the diffusion model and the encoding module together on data pairs for 20 epochs. This can be interpreted as a warm-up for the diffusion model and the encoding module to extract meaningful representations of data.
(2) In the second phase, we include all modules for training, except for solution functions. We consider $p({\tilde{e}}_{i}|e)$ to be a uniform probability density. The framework is trained in this phase for 50 epochs.
(3) We include solution functions and train the whole framework with the proposed loss for 50 epochs.

We find out that considering our data generation process, including the fourth training phase of Brehmer et al. [14] has no impact on the model’s performance. Consequently, we choose to disregard it in our analysis. We use the loss in Equation (3) as the objective function and consider the coefficient of the regularization term $L_{entropy}$ to be 1. Therefore, our overall loss function is then given by $L=L_{model}+L_{entropy}$.

### Appendix C.2. Architectures and Hyperparameters

We train the model for 120 epochs and use the learning rate of 3e-4 with a batch size of 64. β is initially set to 0 and increased to 1 during training. The noise encoder is considered Gaussian, with the mean and standard deviation parameterized as an MLP with two hidden layers and 64 units each and ReLU activation functions. The solution functions are implemented as affine transformations, where the slope and offset are learned from pre-intervention noise encodings. These functions utilize the same architecture as the noise encoder for learning the slope and offset parameters. The architecture of the score function of the diffusion model is based on NCSN++ architecture [53] with the same set of hyperparameters used for the CIFAR-10 dataset. As the input *x* is 16-dimensional and the score model follows a convolutional architecture, we reshape the input into a 4×4 format and then feed it into the diffusion model. Furthermore, in the forward SDE, $\sigma_{min}$ and $\sigma_{max}$ are set to 0.01 and 50, respectively.
